# Supplementary figures and images for: BudFinder: A Masked Auto-Encoder Vision Transformer Framework for Yeast Budding Detection
Source: bioRxiv. 2025 Nov 5:2025.11.04.686463. Preprint. [Version 1] doi: 10.1101/2025.11.04.686463 (PMC12637452; doi:10.1101/2025.11.04.686463)

## Supplemental Figures

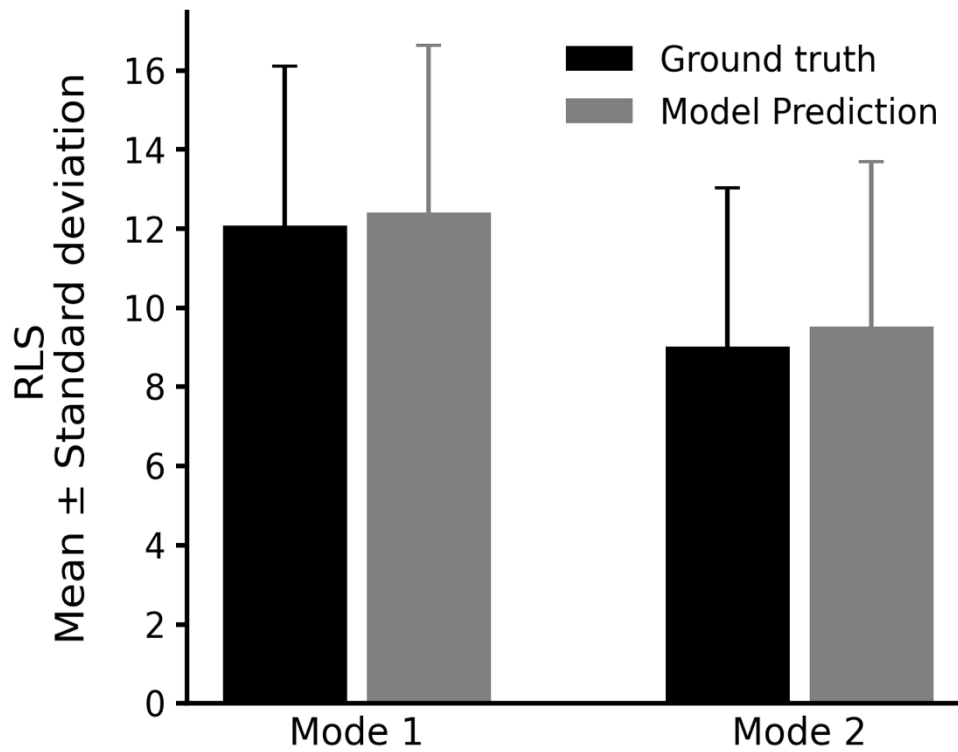

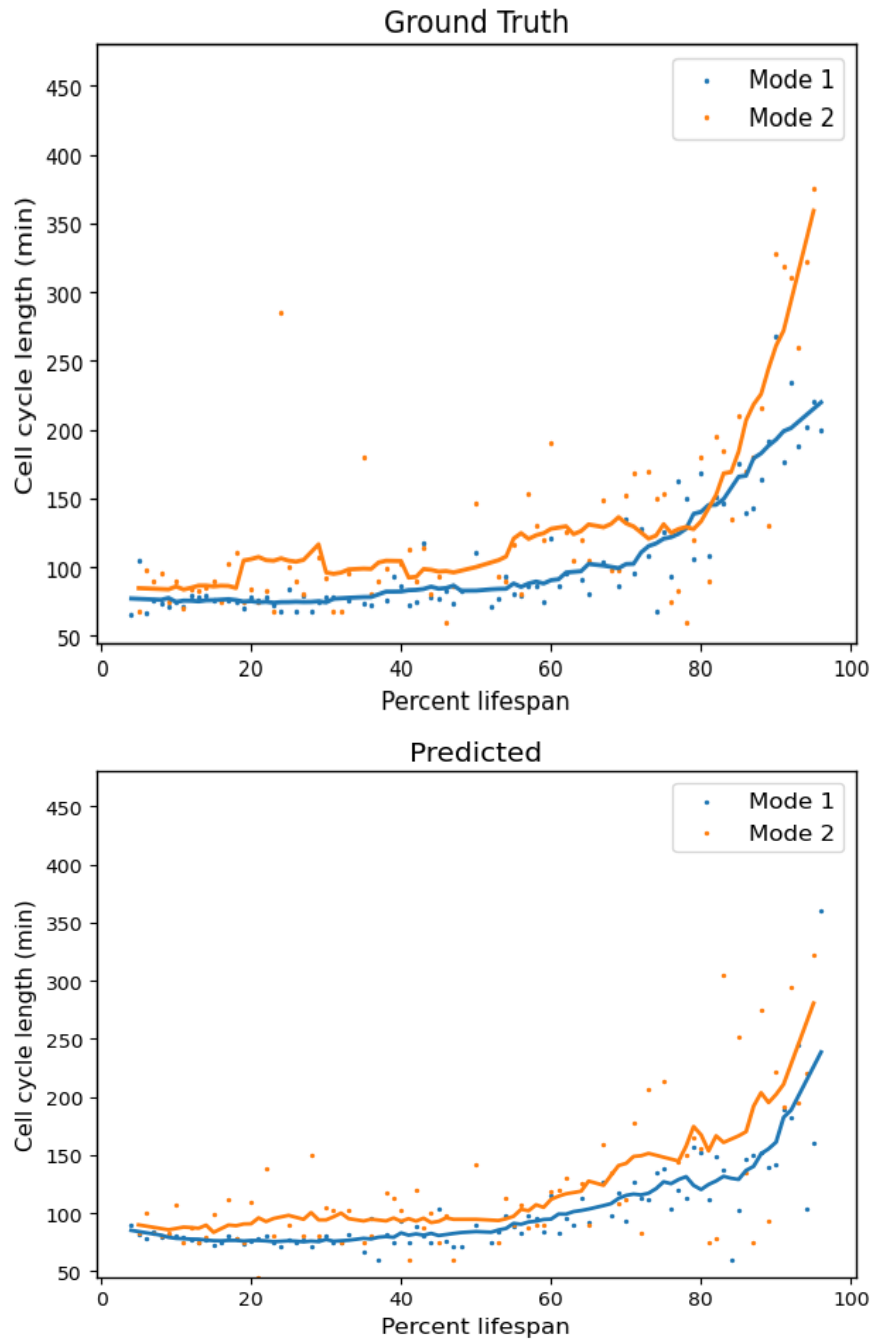

Supplement: 1 [file NIHPP2025.11.04.686463v1-supplement-1.pdf]
